# RNA interference-mediated silencing of VEGF and bFGF suppresses endostatin secretion in pancreatic carcinoma cells

**DOI:** 10.3892/ol.2013.1102

**Published:** 2013-01-02

**Authors:** CHANGQING YAN, CHUAN WANG, MEI DONG, SANGUANG LIU, CHENG QI, YUPEI ZHAO

**Affiliations:** 1 Department of Heptobiliary Surgery, The Second Hospital of Hebei Medical University, Shijiazhuang 050000;; 2 Department of Pharmacology, Hebei Medical University, Shijiazhuang 050017;; 3 Department of Surgery, The Affiliated Hospital of Hebei University of Science and Technology, Shijiazhuang 050018;; 4 Department of General Surgery, Peking Union Medical College Hospital, Peking Union Medical College, Chinese Academy of Medical Science, Beijing 100730, P.R. China

**Keywords:** pancreatic carcinoma, bFGF, VEGF, siRNA, endostatin

## Abstract

Pro-angiogenic factors [vascular endothelial growth factor (VEGF) and basic fibroblast growth factor (bFGF)] and anti-angiogenic factors (endostatin) play important roles in the progression of pancreatic cancer. The purpose of the present study was to investigate the knockdown effect by either VEGF or bFGF siRNA on the expression and secretion of endostatin in pancreatic carcinoma cells. Pancreatic carcinoma cell lines (sw1990, Panc-1 and PCT-3) were treated with VEGF and bFGF siRNA. The expression of VEGF, bFGF and endostatin in pancreatic carcinoma cell lines was determined by reverse transcription-polymerase chain reaction (RT-PCR) and western blot analysis. Secretion of endostatin was measured by enzyme-linked immunosorbent assay (ELISA). bFGF and VEGF siRNA significantly reduced the expression of bFGF and VEGF mRNA, respectively, but did not affect mRNA and protein expression of endostatin in pancreatic carcinoma cell lines. However, secretion of endostatin in PCT-3, Panc-1 and sw1990 cells was significantly inhibited by bFGF and VEGF siRNA. This study demonstrated that pro-angiogenic factors (VEGF and bFGF) differentially modulate expression and secretion of anti-angiogenic factors (endostatin). This result may have important implications in the anti-angiogenesis therapy in pancreatic cancer.

## Introduction

Previous studies have shown that expression of angiogenic growth factors have a close correlation in the progression of pancreatic cancer (1,2). Pancreatic cancer cells produce multiple angiogenic growth factors. They are, therefore, believed to be important sources of those factors. Expression levels of angiogenic growth factors are likely to be closely related to pancreatic cancer cell proliferation and invasion. Different angiogenic growth factors may modulate each other (3,4), particularly in cases where both pro-angiogenic and anti-angiogeneic factors are involved. Vascular endothelial growth factor (VEGF) and basic fibroblast growth factor (bFGF) are by far the most important pro-angiogenic growth factors (5,6), while endostatin has been found to be the strongest anti-angiogenic factor (6,7). In order to further study the modulatory effects between these factors, sw1990, PCT-3 and Panc-1 pancreatic cancer cell lines were infected with VEGF or bFGF siRNA, respectively. We found that VEGF and bFGF siRNA significantly inhibited the secretion of endostatin in PCT-3 and sw1990 cells. This study provided a basis to establish anti-angiogenesis therapy in patients with pancreatic cancer. The study was approved by the ethics committee of Hebei Medical University (Shijiazhuang, China).

## Materials and methods

### Materials

Three human pancreatic cancer cell lines sw1990, Panc-1 and PCT-3 were obtained from the laboratory of General Surgery, Beijing Concord Hospital (China). VEGF siRNA, bFGF siRNA, control siRNA (Fluorescein Conjugate) and siRNA transfection medium were purchased from Santa Cruz Biotechnology, Inc (Santa Cruz, CA, USA). Lipofectamine 2000 and the total RNA Extraction kit were obtained from Invitrogen (Carlsbad, CA, USA). Endostatin ELISA kit was obtained from R&D (Minneapolis, MN, USA). Polyclonal endostatin antibody was obtained from Abcam (Cambridge, MA, USA). Goat-anti-rabbit IgG-HRP was obtained from Santa Cruz Biotechnology, Inc.

### siRNA transfection in pancreatic cancer cell lines

In preliminary experiments, sw1990, Panc-1 and PCT-3 cell lines were transfected with 2, 4, 6, 8 and 10 *μ*l control siRNA (Fluorescein Conjugate), respectively. Lipofectamine 2000 (2.5, 5 and 6 *μ*l) were used to detect transfection efficiency. Before transfection, each type of cancer cell (2×10^5^ per well) was seeded in six-well plates with 2 ml antibiotic-free 1640 medium (10% FBS), at 37°C with 5% CO_2_. When cells reached 60–80% confluence, cells were transfected with a mixture of siRNA and Lipofectamine 2000 which had been incubated at room temperature for 30 min. To determine transfection efficiency, cells were visualized 24 h after transfection by fluorescence microscopy.

### RNA isolation and RT-PCR

Total RNA was isolated from the adult mouse heart. In brief, cells were collected, lysed, and processed for total RNA isolation at 4°C using an RNeasy Plus Mini Kit (Qiagen, Valencia, CA, USA). The concentration of total RNA in each sample was determined using a Nanodrop ND-1000 Spectrophotometer (Thermo Scientific, Wilmington, DE, USA). The integrity of the extracted RNA was confirmed by electrophoresis under denaturing conditions. RT-PCR was performed using iScript cDNA Synthesis Kit (Bio-Rad, Hercules, CA, USA) for the synthesis of a single-stranded cDNA library. PCR reactions were performed using a Bio-Rad PCR machine. The following primers were used: VEGF: 5′-AGCTACTGCCATCCAATCGC-3 ′, 5′-GGCGAATCCAATTCCAAGAG-3′; bFGF: 5′-AGCGGCTGTACTGCAAAAAC-3′, 5′-CCCAGGTCC TGTTTTGGAT-3′; Endostatin: 5′-CTCAATGCAGAGCAC GATGT-3′, 5′-TGTTCTCAGGCTCTGAGGGT-3′; β-actin: 5′-GGCGGCACCACCATGTACCCT-3′, 5′-AGGGGCC GGACTCGTCATACT-3′. PCR products were visualized on 1.5% agarose gel and pictures were taken under UV lamp camera. The relative ratio was calculated using the formula: (A) = A_target gene_ / A_β-actin_.

### Enzyme-linked immunosorbent assay (ELISA) determination of endostatin

Culture medium was collected after treatment with different siRNAs or under control conditions. Endostatin concentrations were determined according to ELISA kit manual (R&D Systems). Each experiment was performed in triplicate.

### Immunoblotting

Cell lysates were prepared by directly extracting cells in lysis buffer containing 150 mM NaCl, 50 mM Tris-HCl, 1% Triton, 0.5% NP40 and protease inhibitor cocktail (Roche, Mannheim, Germany). Following centrifugation at 10,000 rpm for 10 min, supernatants were collected. Protein concentration was quantified using a BCA Protein Assay Kit (Thermo Fisher Scientific, Rockford, IL, USA). Approximately 20 *μ*g protein was dissolved with 4X LDS sample buffer (Invitrogen) and separated on NuPAGE 4–12% Bis-Tris gels (Invitrogen). The blots were visualized by enhanced chemiluminescence and images were captured using a Kodak Image Station 4000 R and quantified using Kodak MI SE software.

### Statistical analyses

Each experiment was performed in triplicate and the results are presented as mean ± standard error. The statistical significance of differences between groups was assessed using either a one-way ANOVA or two-tailed Student’s t-test with SPSS (Chicago, IL, USA) 13.0 software. P<0.05 was considered to indicate a statistically significant result.

## Results

### Effects of bFGF and VEGF siRNA interference on bFGF mRNA expression in different pancreatic cell lines

**Figure 1:**
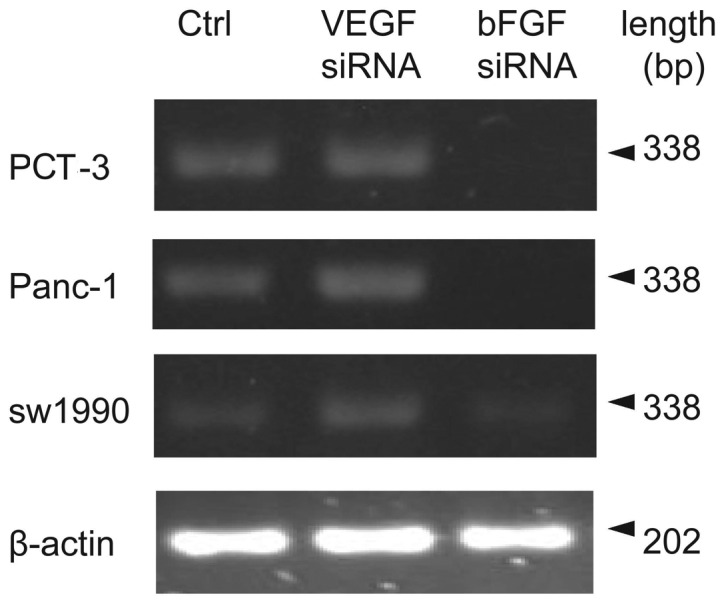
bFGF mRNA expression in different pancreatic cell lines following VEGF and bFGF siRNA treatment, respectively. The representative electrophoresis images are shown.

bFGF or VEGF siRNA was transfected into PCT-3, Panc-1 and sw1990 cell lines. These are stable pancreatic cancer cell lines that express bFGF and VEGF. Using RT-PCR, it was shown that bFGF siRNA but not VEGF siRNA significantly reduced the bFGF mRNA amount in the cell lines tested (P<0.01, compared to control), as shown in Fig. 1 and Table I. Notably. bFGF mRNA expression was increased following VEGF siRNA treatment, especially in Panc-1 cell line (P<0.05, compared to control).

### Effects of bFGF and VEGF siRNA interference on VEGF mRNA expression in different pancreatic cell lines

**Figure 2:**
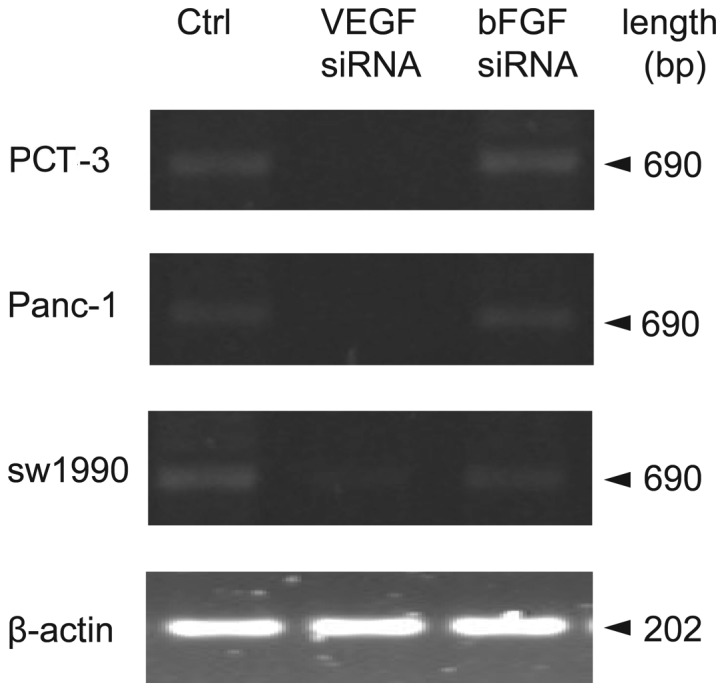
VEGF mRNA expression in different pancreatic cell lines following VEGF and bFGF siRNA treatment, respectively. The representative electrophoresis images are shown.

To test the knockdown effects of both types of siRNA on expression level of VEGF, a similar strategy to detect VEGF expression was used. RT-PCR results (Fig. 2 and Table II) showed bFGF siRNA did not change VEGF mRNA expression in PCT-3 Panc-1 or sw1990 cells (P>0.05, compared to control). However, VEGF mRNA expression was significantly reduced in the three pancreatic cell lines after VEGF siRNA treatment (P<0.05, P<0.01 compared to control).

### Effects of bFGF and VEGF siRNA interference on endostatin mRNA and protein expression in different pancreatic cell lines

**Figure 3:**
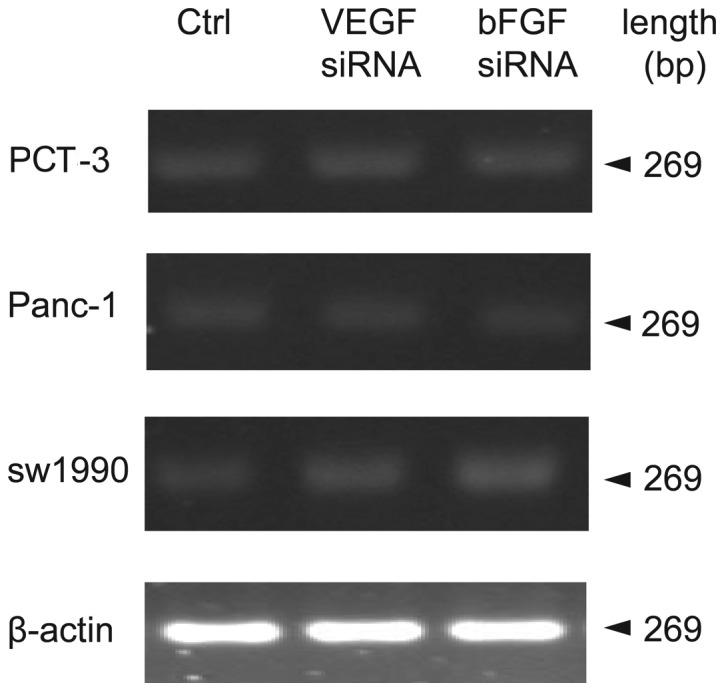
Endostatin mRNA expression in different pancreatic cell lines following VEGF and bFGF siRNA treatment, respectively. The representative electrophoresis images are shown.

**Figure 4:**
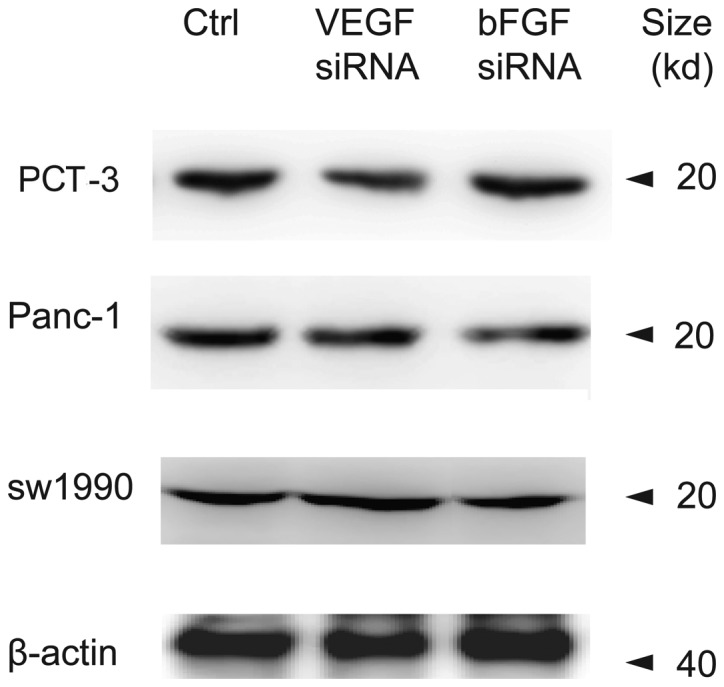
Endostatin protein expression in different pancreatic cell lines following VEGF and bFGF siRNA treatments, respectively. The representative western blot results are shown.

Previous data showed that bFGF or VEGF siRNA was able to dramatically reduce mRNA expression of bFGF or VEGF, respectively. In order to determine the relationship between bFGF, VEGF and endostatin, endostatin mRNA expression was measured by RT-PCR following knockdown of bFGF and VEGF. As shown in Fig. 3 and Table III, neither bFGF nor VEGF siRNA changed endostatin mRNA expression in PCT-3, Panc-1 and sw-1990 cells (P>0.05, compared to control). To further determine whether bFGF siRNA or VEGF siRNA affected endostatin protein expression, western blot assay was employed and results (Fig. 4) showed that neither VEGF nor bFGF siRNA changed endostatin protein expression levels in the cell lines studied.

### Effects of bFGF and VEGF siRNA interference on endostatin concentration in culture supernatant in different pancreatic cell lines

Previous studies showed that the level of exocrine endostatin played important biological roles in the micro-environment of pancreatic cancer cells (8). In order to evaluate the influence of knocking down bFGF and VEGF on the secreted endostatin level, an ELISA technique was used to detect endostatin concentrations in the culture supernatant of different pancreatic cell lines with different treatments. Endostatin concentrations were significantly reduced following treatment with bFGF and VEGF siRNA in the cell lines studied (P<0.01, compared to control; Table IV).

## Discussion

Tumor spread and metastasis are based on angiogenesis. Activation and modulation of angiogenesis are dependent on the interaction between pro- and anti-angiogenic factors (9). Several studies have been performed and it has been recognized that different angiogenic growth factors play important roles in the modulation of neovascularization in all types of tumor progression. Anti-angiogenic therapy is, therefore, one of the most important antitumor therapeutic strategies (10). Anti-angiogenic therapy focuses on two approaches: inhibition of pro-angiogenic factors, or enhancement of anti-angiogenic factors. There are some anti-angiogenic compounds under development in clinical trials. Their therapeutic strategy is often focused on a single therapeutic target. Studies on the interaction between pro-angiogenic factors and anti-angiogenic factors are, however, still scarce. Studies on the interaction may provide a strong basis for joint multi-target anti-angiogenic therapy.

As a type of vasculature-lacking tumor, pancreatic cancer shows atypical angiogenesis accompanied by endothelial cell proliferation, and uneven distribution of vascular morphology (11). This suggests that local factors may account for the pancreatic angiogenic mechanism. A large number of studies confirm that expression of many angiogenic factors, such as VEGF, bFGF and endostatin are elevated in the pancreatic cancer tissue (12), suggesting angiogenic factors play critical roles in pancreatic cancer. Our study shows that VEGF and bFGF siRNAs inhibit VEGF and bFGF, respectively, and also modulate endostatin secretion. A previous study showed that the level of exocrine endostatin was higher than that of intracellular endostatin, suggesting endostatin may play a biological role in the microenvironment of pancreatic cancer cells (8). Our study further indicates that siRNAs of VEGF and bFGF dramatically reduce endostatin concentrations in the culture medium without affecting intracellular endostatin mRNA and protein expression. This suggested that the inhibitory effects of VEGF and bFGF siRNA on endostatin secretion may occur following its transcription and translation, and that modulating protein secretion may be its main mechanism. Brammer *et al*(13) confirmed that collagen XVIII is expressed in pancreatic cancer cells, and can be released into the medium. Heljasvaara *et al*(14) showed that particular matrix metalloproteinases (MMPs) can degrade collagen XVIII and generate biological, suggesting that endostatin is hydrolysized from collagen XVIII. Endostatin is expressed differently in variable pancreatic cancer cell lines, which is modulated by TNF-α-dependent elastase. A study by Nilsson *et al*(15) showed that estradiol and tamoxifen regulate endostatin expression via MMP-2/MMP-9 in breast cancer. A previous study (16) showed that the p38MAPK pathway is involved in the modulation of MMP function, suggesting that the MAPK pathway could be critical for VEGF/bFGF biological function. Further research is required to confirm this.

In pancreatic cancer cells, the regulation of angiogenic factor secretion is complicated and this regulation is closely related to tumor progression (17,18). In the processes of pancreatic cancer occurrence, development and metastasis, pro-angiogenic factors and anti-angiogenic factors modulate each other while each factor has its tumor biological functions (19,20). Further research is required to elucidate the interaction mechanism. This will provide novel therapeutic targets for anti-angiogenic treatment of pancreatic cancer and other tumors. In the development of anti-angiogenic treatment strategies, combined therapy for different targets may yield better treatment results, and in-depth study of modulation mechanisms will further improve the joint treatment effects. The correlation between angiogenic factors in different cancer cell lines may not be the same. Therefore, individualized treatment should be considered when developing joint anti-angiogenic treatment programs.

## Figures and Tables

**Table I:** bFGF mRNA relative expression in different pancreatic cell lines following VEGF and bFGF siRNA treatment, respectively.

		PCT-3		Panc-1		sw1990	
Group	n	Mean value	Fold	Mean value	Fold	Mean value	Fold
Control	3	0.42±0.02		0.62±0.03		0.32±0.03	
VEGF siRNA	3	0.50±0.05	1.19	0.81±0.05^a^	1.31	0.36±0.06	1.12
bFGF siRNA	3	0.08±0.01^b^	0.19	0.10±0.04^b^	0.16	0.15±0.03^b^	0.47

a P<0.05,

b P<0.01 vs. control.

**Table II:** VEGF mRNA relative expression in different pancreatic cell lines following VEGF and bFGF siRNA treatment, respectively.

		PCT-3		Panc-1		sw1990	
Group	n	Mean value	Fold	Mean value	Fold	Mean value	Fold
Control	3	0.28±0.02		0.24±0.02		0.31±0.02	
VEGF siRNA	3	0.08±0.01^b^	0.29	0.06±0.01^b^	0.25	0.16±0.04^a^	0.52
bFGF siRNA	3	0.26±0.03	0.93	0.22±0.03	0.92	0.29±0.02	0.94

a P<0.05,

b P<0.01 vs. control.

**Table III:** Relative expression of endostatin mRNA in different pancreatic cell lines following VEGF siRNA and bFGF siRNA treatment, respectively.

		PCT-3		Panc-1		sw1990	
Treatment	n	Mean value	Fold	Mean value	Fold	Mean value	Fold
Control	3	0.32±0.01		0.28±0.04		0.31±0.03	
VEGF siRNA	3	0.30±0.01	0.94	0.26±0.05	0.93	0.34±0.06	1.10
bFGF siRNA	3	0.33±0.03	1.07	0.32±0.07	1.14	0.33±0.04	1.06

**Table IV:** Concentrations of endostatin in the supernatant of different pancreatic cell lines following bFGF and VEGF siRNA treatments, respectively.

Group	n	sw1990 (ng/ml)	PCT-3 (ng/ml)	Panc-1 (ng/ml)
Control	5	2.98±0.10	15.42±0.75	28.61±3.74
VEGF siRNA	5	0.63±0.12^a^	11.07±0.30^a^	13.67±2.25^a^
bFGF siRNA	5	1.68±0.19^a^	10.69±0.14^a^	14.29±3.65^a^

a P<0.01 vs. control.
